# The prevalence of dental developmental anomalies among childhood cancer survivors according to types of anticancer treatment

**DOI:** 10.1038/s41598-022-08266-1

**Published:** 2022-03-16

**Authors:** Elinor Halperson, Vered Matalon, Gal Goldstein, Shirly Saieg Spilberg, Karin Herzog, Avia Fux-Noy, Aviv Shmueli, Diana Ram, Moti Moskovitz

**Affiliations:** 1 Faculty of Dental Medicine, Hebrew University of Jerusalem, Jerusalem, Israel; 2 Department of Pediatric Dentistry, Hadassah Medical Center, P.O.Box 12272, 9112102 Jerusalem, Israel; 3 Faculty of Medicine, Hebrew University of Jerusalem, Jerusalem, Israel; 4 Department of Pediatric Hematology – Oncology, Hadassah Medical Center, Jerusalem, Israel

**Keywords:** Cancer, Cell biology, Developmental biology, Diseases, Health care, Medical research, Oncology, Risk factors

## Abstract

Survival following childhood cancer has increased considerably. In an observational cross-sectional study, we assessed the prevalence of dental developmental anomalies (DDA) among childhood cancer survivors according to types of anticancer treatment. Permanent teeth were examined clinically and radiographically in 121 adolescents with a history of childhood malignancies, to identify DDA, namely hypomineralization or hypoplasia, microdontia, root changes and hypodontia**.** DDA were observed in 56/121 individuals (46%), in 309/3388 teeth (9%). Hypomineralization or hypoplasia of enamel appeared in 21 (17%) patients. Altered root development appeared in 26 patients and hypodontia affected 13 (10%). Dental anomalies were observed in 36 (43%) individuals who received chemotherapy and not radiation, in 20 (52%) who received radiotherapy, and in 15 (60%) of those who received head and neck radiotherapy. Among patients who received only chemotherapy, young age (6 years or younger) was associated with a higher number of malformed teeth. In conclusion, antineoplastic treatment that combines chemotherapy and radiotherapy appears to increase the risk of DDA. Radiation to the head and neck area was shown to particularly increase the risk of DDA. No specific chemotherapy agent was found to be associated more than the others with DDA.

## Introduction

Current multimodality therapies have improved the survival of patients with childhood cancer. Consequently, research nowadays focuses on the long-term quality of life of these survivors^1,2^, and on the increased risks for various health problems resulting from their childhood cancer or its treatment. Some complications of childhood cancer only become apparent later in life^3,4^. Long-term systemic complications can affect children's general growth and development, and impair their reproductive, respiratory, cardiovascular, skeletal, nervous and endocrine systems^5,6^.

Some oral manifestations may present shortly after inception of cytotoxic and radiation treatment, and some may only become apparent years or even decades after treatment^3^. Amongst other side effects, acute oral effects may include mucositis, bleeding, taste alterations, secondary infections, salivary gland dysfunction, periodontal conditions, trismus, osteoradionecrosis and neurotoxicity^5–12^. Late oral phenomena include exacerbated dental caries, temporomandibular dysfunction, osteoradionecrosis, cranio-dental development, dental developmental anomalies (DDA) and oral graft versus host disease^1,6–12^. Children are particularly vulnerable to the harmful effects of radiotherapy and chemotherapy. A new field in oncology, *'survivorship care'*, focuses on the identification, treatment and prevention of long-term side effects.

Morphogenesis and calcification of teeth begin in utero and continue for 14–15 years; the process is ongoing and complex. Permanent incisors and first permanent molars begin to mineralize around the time of birth but mineralization of permanent dentition is often completed only years later^10,11^. Aberrations in initiation and proliferation of the teeth typically result in failure of tooth development, while insults during histodifferentiation lead to abnormal structure of enamel and dentin. Disturbances during morphodifferentiation can cause abnormal shape and size of teeth^9^. The continuation of severe or long-term upsets may damage root formation, thus leading to a shortened or tapered root. Root development plays a dominant role in the eruption; disturbance to a tooth root might impair tooth eruption and occlusion^9^.

First signs of dental disturbances can be expected within one or two years of anticancer treatment^9^. Reported abnormalities include hypodontia (missing teeth), microdontia (the formation of small teeth), damage to root development (premature closure of the apex, tapering roots with apical constriction, root stunting and V-shaped degenerated root), hypoplasia and hypomineralization (including damage to the enamel structure, resulting in incomplete calcification), over-retention of primary teeth, impaction, premature eruption, malocclusion, decreased temporomandibular joint mobility, trismus and facial deformities^10–20^.

While the literature describes late dental side effects in adults who underwent anticancer treatment during childhood, the effects of particular treatments on dental defects have not been described. Therefore, the purpose of this study was to examine and distinguish dental defects according to type of anticancer treatment (chemotherapy, radiotherapy, surgery), type of chemotherapy treatment, disease type and age during treatment. This information could help identify the children treated for cancer who are at the greatest risk for future dental problems**.**

## Methods

This observational cross-sectional study was conducted and documented according to The Strengthening the Reporting of Observational Studies in Epidemiology (STROBE) statement. The data for this study were collected and the analyses performed during 2021.

### Study population

The study population consisted of individuals who underwent annual general examinations at the survivorship care clinic of Hadassah Hebrew-University Medical Center, Jerusalem, Israel during 2017–2019, including full oro-dental examinations at the Department of Pediatric Dentistry. Eligibility criteria included anticancer treatment at age under 18 years at the Department of Pediatric Hematology–Oncology, Hadassah and at least 7 years old on the day of the dental examinations.

### Medical variables

The demographic data recorded included the age at diagnosis, the age at the dental examination and gender. The medical history accessed comprised primary diagnosis, other medical conditions, the type of therapy or therapies applied (chemotherapy, radiotherapy, surgical excision, bone marrow transplantation (BMT)), systemic late complications, the chemotherapy agents used, the dates of chemotherapy sessions and complications after chemotherapy. Chemotherapy medications were divided into eight groups, classified according to their mechanisms of action.

### Dental variables

Using a dental mirror and explorer, permanent teeth were examined for dental caries and dental DDA under the artificial light of the dental unit. The radiographic dental examination included a set of two bitewings and a panoramic x-ray if indicated. The DMFT index scoring system was used to permit the calculation of caries. Decayed (D), Missing (M), Filled (F) per teeth (T) according to World Health Organization criteria. The presence of DDA was classified into five major groups: no disturbance identified, hypomineralization or hypoplasia, microdontia, root changes, and an absent tooth bud categorized as hypodontia. Hypomineralization or hypoplasia was defined as the developmental anomaly of enamel mineralization. We included every visualized opacity (demarcated or diffuse) that was deemed developmental according to the location and appearance. Microdontia was defined as a change in tooth size by visual judgment when the size of a tooth crown was 50% of the size considered “normal”^18,22^. A root change was defined as a change in root size or shape by visual judgment of the X-rays, when the size of a root was 50% or less of the size considered “normal”.

### Ethical considerations

The study protocol was approved by the Institutional Human Subjects Ethics Committee of Hadassah Medical Organization, Jerusalem, Israel (0004-16-HMO date of approval: April 4, 2017). All the methods were carried out in accordance with relevant guidelines and regulations^19^. Informed consent was obtained from the participants, or from the parents or legal guardians of those under age 18 years.

### Statistical methods

The data were analyzed using statistical software (Stata, V 12.1; Stata, College Station, TX, USA). Descriptive statistics, including numbers and percentages of patients were tabulated for demographic and clinical characteristics. Chi-squared and Fisher's exact tests were utilized to examine associations between the categorical variables and treatment groups. The t-test was utilized to examine associations between the continuous variables (DMFT) and treatment groups. The data were stratified by age, and an analysis compared outcomes between participants aged ≤ 6 years and those aged > 6 and ≤ 12 years. For these analyses, a *p* value less than 0.05 was considered statistically significant.

## Results

Of 131 childhood cancer survivors who were referred to the Department of Pediatric Dentistry for full oro-dental examinations, 121 met the study eligibility criteria. Their mean age was 7.1 years (range 0.1–17.7), and they underwent dental evaluations at a mean age of 15.9 (range 12.0–23.1) years. Table 1 presents the demographic characteristics of the patients, the types of cancer and types of treatment. The underlying diseases were leukemia\lymphoma in 53 (45%) patients, solid tumors in 35 (29%) and other hematological conditions leading to BMT in 31 (26%). Most patients (83, 69%) had received chemotherapy without radiotherapy. Thirty-eight (31%) had received radiation therapy only or in combination with chemotherapy. Fourteen (12%) of the cohort had received total body irradiation (TBI) 12 Gray and 15 (13%) radiation to the head and/or neck area (range of 27–70 Gray). The remaining nine patients had received radiotherapy to other areas (range of 30–70 Gray). Thirty percent of the cohort had undergone BMT.

**Table 1:** Patient characteristics.

Variable	# of patients (%)
**Gender**	*N* = 121
Male	76 (63%)
Female	45 (37%)
**Diagnosis category (1)**	*N* = 121
Acute lymphocytic leukemia	27 (22%)
Acute myelocytic leukemia	10 (8%)
Non-Hodgkin lymphoma	9 (7%)
Hodgkin lymphoma	7 (6%)
Sarcoma	17 (14%)
Neuroblastoma	14 (12%)
Other solid tumors	4 (3%)
Hematological condition	31 (26%)
Other	2 (2%)
**Diagnosis category (2)**	*N* = 119
Leukemia and lymphoma	53 (45%)
Solid tumor	35 (29%)
Hematological	31 (26%)
**Treatment**	*N* = 121
Chemotherapy only	83 (69%)
Any radiation (chemotherapy and radiation therapy or radiation therapy only)	38 (31%)
**Radiotherapy**	*N* = 121
None	83 (69%)
Total body irradiation	14 (12%)
Head/neck	15 (13%)
Other	9 (7%)
**Surgical treatment**	*N* = 121
No	102 (84%)
Yes	19 (16%)
**Bone marrow transplantation**	*N* = 121
No	85 (70%)
Yes	36 (30%)

**Figure 1:**
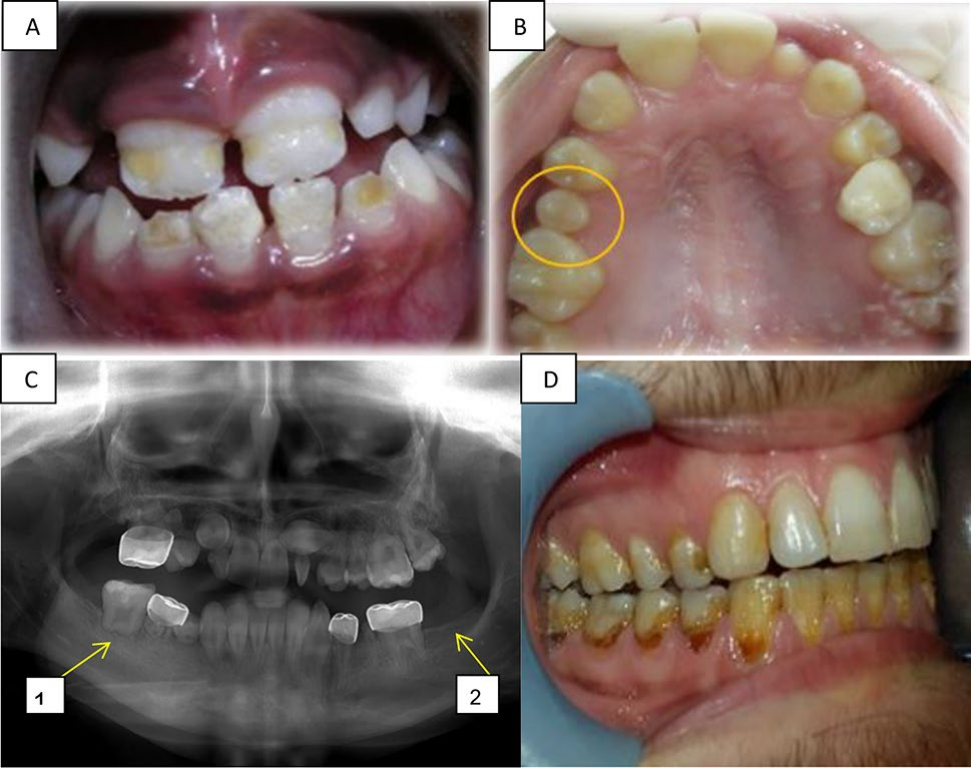
Long term dental effects. (**A**) Hypoplasia in the front upper and lower teeth of a girl aged 9 years, treated for ALL at age 3.5 years. (**B**) Microdontia showing the second upper right premolar in a girl age 12 years, treated for neuroblastoma at age 4 years. (**C**) A panoramic radiograph of a 12-year-old boy diagnosed with Burkitt's lymphoma at age 4 years, revealing: C_1_. Altered root development at the first lower right molar, C_2_. Hypodontia of the second lower left molar. (**D**) Radiation caries in a 21-year-old boy treated for neuroectodermal tumor at age 14 years.

Table 2 presents the numbers of participants and the numbers of teeth with each dental anomaly. In total, 56 (46%) patients had at least one DDA, in 309/3388 teeth (9%). Hypomineralization or hypoplasia of enamel presented in 21 (17%) patients (Fig. 1A); and the same number of microdontic teeth presented (Fig. 1B). Altered root development presented in 26 (21%) patients (Fig. 1C1) and hypodontia in 13 (11%) (Fig. 1C2). Table 3 presents DDA according to anticancer treatment modalities. Malformed teeth were detected in 36/83 (43%) patients who had received only chemotherapy, 20/38 (53%) of those who had received radiation, 15/36 (42%) of those who underwent BMT, and 9/15 (60%) of those who had received radiation to the head and/or neck. The age at initiation of oncology treatment ranged from 0 to 18 years. The proportion of patients with malformed teeth was higher among those who initiated treatment at age 6 years or younger (31/55, 56%) than among those who initiated treatment between ages 6 and 12 years (19/43, 44%) (Table 4). In addition, all the types of DDA were more frequent in individuals who initiated anticancer treatment at age 6 years and younger (Table 4).

**Table 2:** The number of children who presented with each dental developmental anomaly, and the number of teeth involved.

Type of malformation	#children *N* = 121	#teeth *N* = 3388
None	65 (54%)	3079 (91%)
Hypocalcification or hypoplasia	21 (17%)	62
Microdontia	21 (17%)	57
Root changes	26 (21%)	160
Hypodontia	13 (11%)	30
Any malformation	56 (46%)	309 (9%)

**Table 3:** Dental developmental anomalies according to anticancer treatment modalities.

	Chemotherapy only *N* = 83	Any radiation *N* = 38	BMT *N* = 36	Head/neck radiation *N* = 15
Categorical variables	*N* (%)	*N* (%)	*N* (%)	*N* (%)
Any malformed teeth	36 (43%)	20 (53%)	15 (42%)	9 (60%)
The number of malformed teeth				
0	47 (57%)	18 (47%)	21 (58%)	6 (40%)
1	7 (8%)	5 (13%)	4 (11%)	2 (13%)
2	7 (8%)	3 (8%)	0	2 (13%)
3	8 (10%)	2 (5%)	5 (14%)	1 (7%)
4	8 (10%)	0	3 (8%)	0
> 5	6 (7%)	10 (26%)	3 (8%)	4 (27%)
The type of malformation (> 1 tooth with malformation)				
Hypocalcification or hypoplasia	11 (13%)	10 (26%)	5 (14%)	5 (33%)
Microdontia	16 (19%)	5 (13%)	7 (19%)	3 (20%)
Root changes	15 (18%)	11 (29%)	5 (14%)	4 (27%)
Hypodontia	9 (11%)	4 (11%)	4 (11%)	2 (13%)
DMFT, mean (SD)	5.93 (5.73)	8.37 (6.88)	6.67 (6.85)	7.93 (5.46)

BMT, bone mineral transplantation; DMFT, decayed, missing, and filled teeth; SD, standard deviation.

**Table 4:** Dental anomalies in children who received anticancer therapies, according to the age of initiation of treatment.

Type of malformation	≤ 6 years *N* = 55	> 6 ≤ 12 years *N* = 43	*P*-value *Chi-squared test utilized
Total number of children with any malformation	31 (56%)	19 (44%)	0.231
Hypocalcification or hypoplasia	8 (15%)	10 (23%)	0.269
Microdontia	18 (33%)	3 (7%)	0.002*
Root changes	15 (27%)	9 (21%)	0.469
Hypodontia	11 (20%)	1 (2%)	0.007*
Total number of malformed teeth	4.15 (6.85)	1.67 (3.82)	0.013*
DMFT	6.07 (6.49)	6.02 (4.70)	0.483

Note that this analysis included only children aged 12 years and younger at the initiation of treatment.

DMFT, decayed, missing, and filled teeth.

The data are presented as *N* (%) or as mean (standard deviation).

Table 5 describes DDA according to eight groups of chemotherapy agents, classified according to their mechanisms of action. Most of the patients (*N* = 46, 38%) had received a combination of three agents. The maximum combination treatment was composed of six agents. Ninety-eight patients had received alkylating agents. The number of malformed teeth and the types of malformation did not differ significantly according to chemotherapy agent. The mean DMFT in all 121 patients was 6.69 (standard deviation [SD] 6.19) (Fig. 1D). The 'D' component was the highest, 4.4 (SD 4.38); next was ‘M’, 0.51 (SD 2.40); and then ‘F’, 1.84 (SD 3.17). The mean DMFT values differed according to treatment: 5.93, 8.37, 6.85 and 7.93 for patients who were treated with chemotherapy only, radiation of any type, BMT and head/neck radiation, respectively (Table 3). Compared to patients who had received only chemotherapy, among those who had received any radiation, the DMFT was higher (*p* = 0.022), as was the total number of malformed teeth (*p* = 0.051). Statistically significant differences were not found in the mean DMFT according to chemotherapy agents (Table 5). For most of the parameters examined, statistically significant differences were not found between males and females. Two statistically significant differences regarding gender were observed: tooth malformation microdontia was more common among females (*p* = 0.037) and decayed teeth were more common among males (*p* = 0.025).

**Table 5:** Dental developmental anomalies and DMFT (decayed, missing, and filled teeth) in childhood cancer survivors, according to chemotherapy agents, classified into 8 groups.

Classification	ATG *N* = 25	Alkylating Agent *N* = 98	DNA Crosslinking Agents *N* = 43	Antimetabolite *N* = 53	Topoisomerase 2 Inhibitors *N* = 67	Hormonal Agents *N* = 36	Tubulin Inhibitors *N* = 67	Miscellaneous *N* = 41
Drugs	*Anti-thymocyte globolin*	*Busulfan Melphalan Treosulfan Cyclophosphamide Dacarbazine Temozolomide Thiotepa Ifosfamide Mitomycin-C Dactinomycin Cytoxan*	*Fludarabine Cisplatin* *Thiotepa Melphalan Carboplatin*	*5-Fluorouracil* *6-Mercaptopurine Capecitabine Cytarabine Floxuridine* *Fludarabine Gemcitabine Hydroxycarbamide Methotrexate Pemetrexed Cytosar-U Thioguanine*	*VP16, Doxorubicin* *Irinotecan Daunorubicin Mitoxantrone Esroubicin Teniposide Amsacrine Topotecan*	*Tamoxifen Aromatase inhibitors Androgens* *High doses estrogen*	*Colchicine vinblastine vincristine vinorelbine* *halichondrins*	*Aspaginase Aclacinomycin Streptozocin Menogaril* *Actinomycin* *Bleomycin* *ATRA* *Bleomycin* *Imatinib* *Desatimb Brentuximab*
Any malformed teeth	11 (44%)	44 (45%)	23 (53%)	25 (47%)	32 (48%)	19 (53%)	32 (48%)	19 (46%)
Type of Malformation (> 1 tooth with malformation)								
Hypocalcification or Hypoplasia)	3 (12%)	16 (16%)	11 (26%)	8 (15%)	12 (18%)	5 (14%)	13 (19%)	5 (12%)
Microdontia	4 (16%)	17 (17%)	8 (19%)	11 (21%)	14 (21%)	9 (25%)	14 (21%)	8 (20%)
Root change	4 (16%)	21 (21%)	9 (21%)	11 (21%)	16 (24%)	11 (31%)	17 (25%)	10 (24%)
Hypodontia	4 (16%)	11 (11%)	6 (14%)	3 (6%)	7 (10%)	2 (6%)	6 (9%)	2 (5)
DMFT	6.12 (6.37)	6.31 (6.23)	7 (5.99)	6.53 (6.88)	5.85 (5.69)	6.94 (6.01)	6.99 (6.27)	6.54 (6.87)
Total number of malformed teeth	2.4 (5.73)	2.55 (5.43)	3.42 (6.96)	2.17 (4.09)	2.37 (4.50)	2.31 (3.21)	2.99 (5.58)	2.61 (5.09)

The data are presented as number (%) or mean (standard deviation).

## Discussion

The main finding of this study is that prevalences of dental anomalies in survivors of childhood cancer differed according to the type of cancer treatment administered. Prevalences were higher among those who had received radiotherapy, and particularly targeted to the head and neck area.

### Prevalences and types of dental anomalies

Treatment of childhood cancer is a success story of modern medicine, in which effective treatments have been identified for previously untreatable diseases. The growing population of child survivors of cancer, as well as young adult survivors, will require considerable attention from the medical and dental community in the decades to come. In our cohort of individuals who had received anticancer therapies, 46% had at least one malformed tooth; and 13% had more than five malformed teeth. Defects in enamel development and alterations in tooth size were equally distributed among the patients (17%). Sixty-two teeth of 21 (17%) patients displayed some form of hypomineralization or hypoplasia. This rate is lower than rates of dental alteration reported by other studies^9,21–29^. Possible reasons for the discrepancy are the wide range of age of our cohort (0–18 years) and the range of cancer type and anticancer therapies. In addition, improved oncology treatments during recent years have become more targeted and confer fewer side effects, including dental side effects. Twenty-one patients (17%) in the current study displayed microdontia, in contrast to 0.8%-1.7%^20^ in healthy populations. Microdontia causes esthetic, functional and occlusal complications, which require professional dental treatment later in life. Alterations of tooth root were the most frequent malformation found in the current study; however, the rate of 21% was low compared to 86% and even 100% in other reports of cancer survivors^20^. In healthy populations, rates of 1.3%–5.6% have been reported^20^. As tooth development is a relatively slow process, DDA may become evident on radiographs only two years after a triggering event. Intensive, repetitive chemotherapy at the time of initial hard tissue formation may cause tooth hypodontia^15,20^, which is the most severe impairment in dental development. Hypodontia affects dental arches, and impairs tooth symmetry, esthetics and function. Hypodontia was seen in 11% of our patients, which compares with 2.8%–10% in healthy populations^22,28^.

### Associations of various anticancer therapies with DDA

Thirty-one percent of our patients who received chemotherapy were treated with radiation in addition. More than half the patients (53%) who received radiation displayed malformed teeth, a higher proportion than among those who received only chemotherapy (43%). This difference concurs with a previous study^27^. Acute damage from chemotherapy seems to be greater when the treatment is combined with head and neck irradiation or TBI, rather than administered as a monotherapy^13^. Notably, among our 15 patients who had received head or neck radiation (25–70 Gray), the prevalence of DDA was higher than among those who had received chemotherapy alone or TBI (12 Gray). This finding concurs with other studies^4,14^.

### Associations of chemotherapy agents with DDA

Many pediatric cancers are treated with a combination of multi-agent chemotherapy to create synergistic and additive effects. In the current study, 46 patients (38%) had received a combination of three chemotherapy agents; the maximum combination treatment comprised six agents. The use of multiple agents makes it difficult to attribute specific influence on odontogenesis to any single agent or therapy^18^, and the odontogenic toxicities induced by individual chemotherapy agents remain obscure^29^. The size of the current cohort was small for evaluating the effects of individual chemotherapeutic agents on dental developmental defects. We suggest that more studies will investigate associations with DDA, of chemotherapy agents according to their mechanisms of action, using the eight categories of chemotherapy agents presented herein.

### Associations of age and gender with DDA

Malformed teeth of all the types examined presented more frequently among children who received anticancer treatment at age 6 years or younger than among older children. Young age also remained a significant factor for the total number of malformed teeth among patients who received chemotherapy only (*p* = 0.001). Several publications suggested that children diagnosed with cancer between ages 3 and 5.5 years exhibited the most severe DDA^11,15,20,22,29^. This is consistent with the initial stage in this age interval, of root formation for all permanent teeth except the second and third molars. Treatment administered during the first 3.5 years of life was more likely to affect the dental lamina and crown formation, and to result in a small tooth^22^. Anticancer therapy administered after age 5 years may still disturb root growth, especially in late developing premolars and permanent second molars. However, by this age, the roots in the early developing teeth have already reached a moderate length, which improves the final result^29^. In several studies, the most extensive DDA (agenesis, microdontia and root anomalies) were reported in children who were treated before ages 5–6 years^1,4,14,22,29^, due to the proliferation of dental stem cells during this period^1,2,25,29^.

Only minor differences were found between boys and girls in the current study. Two significant differences regarding gender were noticed: microdontia was higher among females (*p* = 0.037) and decayed teeth were more prevalent among males (*p* = 0.025).

### Dental caries (DMFT)

The mean DMFT score for the study group was 6.69. This score is much higher than 1.66, which was reported for healthy 12-year-old children in Israel^31^. In the present study, the 'DT' component was the highest. Our findings concur with other reports of higher incidence of caries in children who received antineoplastic therapy^12,15,21,29^. For our patients who received radiotherapy, the DMFT was even higher, 8.37; this compared to a score of 5.93 among patients who received only chemotherapy. Aggressive and extensive caries, commonly known as radiation caries (such as seen in Fig. 1D), have a rapid onset and progression^12^. Radiation caries result from the sequelae of xerostomia, teeth hypomineralization or hypoplasia, and a cariogenic shift in microflora^30^. Teeth demineralization following radiotherapy can be remineralized^30,32^. To avoid the development of osteoradionecrosis following radiotherapy, and to avoid the loss of teeth, dentists should be conservative and try to preserve teeth with endodontic and restorative treatment^12^.

### Limitations

Our study has several limitations that should be considered when interpreting the results. In addition to cancer therapy, other genetic and environmental factors may influence tooth development. We were not able to account for outcomes that could be due to differences between patients in age, the time lapsed from diagnosis and from treatment, and the presentation of chronic health conditions. Moreover, family history, hygiene patterns and socioeconomic status play crucial roles in dental health, and can affect many variables and especially DMFT score. The lack of a control group is a limitation of the study. We focused on the influence of various anti-cancer modalities and medications on DDA. Differences between healthy children and childhood cancer survivors have been described in the literature. Thus, we aimed to understand the anticancer protocols and medication that lead to those differences. Another limitation of the study is the limited size of the patient survivor group that was referred to the Department of Pediatric Dentistry from the survivorship care clinic during the study period and that matched the eligibility criteria. Future studies should include other centers that use similar treatment protocols.

## Conclusion

In our cohort of childhood cancer survivors, combined chemotherapy and radiotherapy, and particularly radiation to the head and neck area, seems to have increased the risk of DDA. The results of this study may help direct physicians to identify childhood cancer survivors at high risk of having DDA. This highlights the importance of dental care for individuals who received oncology treatment at a young age (0–6 years), particularly if combined with radiotherapy, and especially in the head or the neck region. No specific chemotherapy agent was found to be associated more than the others with dental side effects. The particular importance of our study in its cross-sectional examination of variables can be enhanced in large centers. This can help hone the results to identify risks of adverse dental effects for specific treatments and at particular stages of child development, and establish international guidelines for follow-up and treatment.

## Abbreviations

BMT: Bone marrow transplantation
DDA: Dental developmental anomalies
DMFT: Decayed, missing and filled teeth
SD: Standard deviation
TBI: Total body irradiation
